# A novel inflammation‐based nomogram system to predict survival of patients with hepatocellular carcinoma

**DOI:** 10.1002/cam4.1787

**Published:** 2018-09-27

**Authors:** Jinbin Chen, Aiping Fang, Minshan Chen, Yiminjiang Tuoheti, Zhongguo Zhou, Li Xu, Jiancong Chen, Yangxun Pan, Juncheng Wang, Huilian Zhu, Yaojun Zhang

**Affiliations:** ^1^ State Key Laboratory of Oncology in South China, Collaborative Innovation Center for Cancer Medicine Sun Yat‐sen University Cancer Guangzhou China; ^2^ Department of Hepatobiliary Oncology Sun Yat‐sen University Cancer Center Guangzhou China; ^3^ Department of Nutrition, School of Public Health Sun Yat‐sen University Guangzhou China

**Keywords:** hepatocellular carcinoma, inflammation‐based score system, nomogram, prognostic value

## Abstract

**Background and Aim:**

The existed staging systems were limited in the accuracy of prediction for overall survival (OS) of hepatocellular carcinoma (HCC) patients. The aim of this study is to establish a novel inflammation‐based prognostic system with nomogram for HCC patients.

**Methods:**

A prospective cohort of patients was recruited and assigned to the training cohort (n = 659) and validation cohort (n = 320) randomly. Different inflammation‐based score systems were evaluated to select the best one predicting overall survival (OS). The inflammation‐based score system with the highest predicting value and the parameters best reflecting tumor burden identified by multivariate analysis were selected to construct a novel predicting nomogram system. The predictive accuracy and discriminative ability of the nomogram were evaluated by concordance index (C‐index) and calibration curve and compared with conventional staging systems.

**Results:**

With a highest C‐index and areas under the receiver operating characteristic curve (AUC), C‐reactive protein/albumin ratio (CAR) was selected to construct the novel system, along with tumor number, tumor size, macrovascular invasion and extra‐hepatic metastases. The C‐index of the nomogram was 0.813 (95% CI, 0.789‐0.837) in the training cohort and 0.794 (95% CI, 0.756‐0.832) in the validation cohort. The calibration curve for predicting probability of survival showed that the nomogram had a high consistency with follow‐up data. The C‐index of the novel system was higher than other conventional staging systems (*P* < 0.001).

**Conclusions:**

The novel inflammation‐based nomogram, developed from prospectively collected data in the present study, predicted the OS of HCC patients.

## 1 INTRODUCTION

Hepatocellular carcinoma (HCC) is the third leading cause of cancer‐related deaths globally. An estimated 782 500 new liver cancer cases and 745 500 deaths occurred worldwide during 2012, with China alone accounting for about 50% of the total number of cases and deaths.^1^ To predict the overall survival (OS) of the HCC patients, several staging systems were proposed, including Barcelona Clinic Liver Cancer (BCLC),^2^ the American Joint Committee on Cancer (AJCC) seventh edition,^3^ Okuda staging system,^4^ Japan Integrated Staging Score (JIS),^5^ Cancer of the Liver Italian Program (CLIP)^6^ and Chinese University Prognostic Index (CUPI).^7^ Unfortunately, the systems aforementioned were limited in the accuracy of prediction and could not be popularized worldwide. A pragmatic and powerful predicting system based on objective measures is in great need.

Recently, systemic inflammation was reported to have close relationship with malignancy.^8^ Different inflammation‐based scores, mainly calculating the quantitative value of plasma neutrophil count, lymphocyte count, platelet count, albumin level, and C‐reactive protein (CRP) level or the ratio between two indicators, were proposed and to be considered useful in this aspect.^9^, ^10^, ^11^ However, the systemic inflammation alone is not adequate to predict the OS of HCC patients. Adding the systemic inflammation and tumor characteristics together is of great potential to provide an accurate and robust prediction system.

The present study aimed to establish a novel predicting system with nomogram combining the systemic inflammation and tumor burden factors, which give rise to a satisfying prognostic indication to HCC patients. The inflammation‐based prognostic factor was carefully selected from the inflammation systems which had been reported in the previous studies.

## 2 METHODS

### 2.1 Study populations and design

This study population came from a prospective cohort of patients recruited in Sun Yat‐sen University Cancer Center, from September 2013 to March 2016. This study was approved by the Institutional Review Board of Sun Yat‐sen University Cancer Center and conducted in accordance with approved guidelines. It was approved by the Institutional Ethics Committee. All patients were fully informed that their data were to be used for research, and related consent was signed. The patients who met the following criteria were included: (a) Diagnosed with HCC based on the criteria of the European Association for the Study of the Liver.^12^ Pathological diagnosis was required if the clinical diagnosis was not clear. (b) No prior treatment was undertaken. (c) Eastern Cooperative Oncology Group (ECOG) performance status of 0‐2. The patients in the primary cohort were randomly assigned to two groups: a training cohort to establish a predicting system and a validation cohort to confirm the predicting power of the new system, at the ratio of 2:1.

Demographics and clinical data were collected prospectively. Important clinical data included: performance status, underlying liver diseases (hepatitis B virus [HBV]infection, hepatitis C virus [HCV] infection, liver cirrhosis), parameters of liver function (albumin [ALB], alanine transaminase [ALT], aspartate transaminase [AST], and total bilirubin [TBIL] levels, etc), tumor characteristics reported by radiology studies (size, number, macrovascular invasion, and extra‐hepatic metastases), alpha‐fetoprotein (AFP) concentration and systemic inflammation factors (white blood cell [WBC] count, neutrophil count, lymphocyte count, platelet count, and CRP level).

The following staging systems were used to restage the patients subsequently: AJCC 7th edition (2010), BCLC, Okuda, CLIP, CUPI, and JIS.

### 2.2 Following up

The patients were followed up one month after initial treatment and every 3 months thereafter. Surveillance included blood routine test, liver profile, AFP level, and dynamic computed tomography (CT), or magnetic resonance imaging (MRI). CT of the chest, bone scintigraphy or Positron emission tomography CT (PET‐CT) was performed when extra‐hepatic metastases were suspected.

### 2.3 Statistical analysis

Statistical analysis was performed with SPSS 20.0 software (SPSS, Chicago, IL, USA) and R 3.4.3 (https://www.r-project.org/). Student's *t* test was used to compare continuous variables when the data distributed normally. Mann‐Whitney *U* test was used to compare skewed data. And chi‐squared or Fisher's exact test was used for categorical variables. A *P* value <0.05 was considered to indicate statistical significance.

The inflammation‐based prognostic scores frequently reported in previous studies were chosen after literature review and calculated accordingly. The scores were further translated into rank variable referring to the original article definition or based on the receiver operating characteristic (ROC) curve established with the data from the training group. Optimal cut‐offs of ROC curve were identified by calculating the Youden index. The predictive value of the inflammation‐based score system was evaluated by the C‐index in R and area under ROC (AUC) in SPSS. The system with the highest predicting capacity was chosen to establish a novel predicting system.

Overall survival was the primary endpoint of the analysis, defined as the time from diagnosis to death or to the last follow‐up date in patients whose data were censored.OS was demonstrated by Kaplan‐Meier analysis, and the curves were compared by the log‐rank test. Univariate and multivariate analyses were performed using the Cox regression model and the associated 95% confidence interval (CI) was calculated. A nomogram was formulated based on the results of multivariate analysis and by the package of rms in R. The C‐index and calibration curve were derived based on regression analysis. Comparisons between the nomogram and other staging systems were performed with the rcorrp.cens in Hmisc in R and were evaluated by the C‐index.^13^ The nomogram was applied in validation group to confirm the predicting value, which statistical methods were the same as those used in the training group. And subgroups analysis stratified by different initial treatments was performed to test the predicting ability of the novel system also.

## 3 RESULTS

### 3.1 Baseline characteristics of patients

After excluding two patients with PS score of more than 2, a total of 979 consecutive patients in the prospective cohort met the inclusion criteria and were included in the primary pooled cohort. The patients were then assigned to the training cohort (n = 659) or the validation cohort (n = 320) randomly by the “select cases” function of SPSS. The baseline characteristics of the primary pooled cohort, the training cohort, and the validation cohort were shown in Table 1. Most patients were in the ECOG performance status of 0 (607/979, 62.00%) and 1 (367/979, 37.49%). Nearly all of the patients had preserved liver function with Child‐Pugh A level (965/979, 98.57%). The numbers of patients with tumor smaller than 3 cm, between 3 and 5 cm and larger than 5 cm were 239, 209 and 531, respectively. The number of patients with single tumor was 608 (608/979, 62.10%). Multiple tumors were observed in the remaining 371 patients (371/979, 37.90%). Macrovascular invasion (167/979, 17.06%) and extra‐hepatic metastases (109/979, 11.13%) could be identified in part of the patients. Liver cirrhosis was identified in 628 patients (64.15%). Among the whole primary cohort, 571 patients (571/979, 58.3%) received radical therapies as the initial treatment, including 475 cases (475/979, 48.5%) liver resection and 96 cases (96/979, 9.8%) radiofrequency ablation. The remaining 408 patients (408/979, 41.7%) received palliative therapies and most of them (367/979, 37.5%) received transcatheter arterial chemoembolization (TACE) as initial treatment. These characteristics were not significantly different between the training cohort and validation cohort.

**Table 1:** Baseline characteristics of the patients

Characteristics	Primary cohort	Training group	Validation group	*P* value
No. of cases	979	659	320	
Sex (male/female)	767/112	580/79	287/33	0.440
Age (years, <65/≥65)	839/140	558/101	281/39	0.188
ECOG (0/1/2)	607/367/5	405/250/4	202/117/1	0.752
HBV (+/−)	884/95	595/64	289/31	0.990
HCV (+/−)	16/963	8/651	8/312	0.137
Child‐Pugh class (A/B/C)	965/13/1	650/8/1	315/5/0	0.711
MELD score (range)	4.52 (−5 to 29)	4.42 (−5 to 29)	4.81 (−4 to 16)	0.252
Number of tumors (Single/Multiple)	608/371	406/253	202/118	0.646
Size of tumors (cm, <3/3‐5/＞5)	239/209/531	162/137/360	77/72/171	0.829
Macrovascular invasion (Y/N)	167/812	116/543	51/269	0.516
Extra‐hepatic metastases (Y/N)	109/870	65/594	44/276	0.070
Cirrhosis (Y/N)	628/351	424/235	204/116	0.857
AFP (ng/mL; ≤400/>400)	622/357	424/235	198/122	0.452
White blood cells (×10^9^/L)	6.10 (1.43 to 21.4)	6.07 (1.43 to 21.4)	6.14 (2.33 to 18.8)	0.199
Platelet count (×10^9^/L)	177.0 (27.3 to 582.0)	178.0 (29.0 to 582.0)	175.25 (27.3 to 562.9)	0.463
Hemoglobin (g/L)	146.0 (35.0 to 230.0)	146.0 (35.0 to 230.0)	146.2 (54.0 to 199.0)	0.915
Serum ALT (U/L)	38.2 (9.4 to 672.5)	39.0 (9.8 to 450.8)	37.8 (9.4 to 672.5)	0.697
Serum AST (U/L)	39.5 (11.3 to 767.5)	39.5 (11.3 to 767.5)	38.1 (14.7 to 614.7)	0.658
Total bilirubin (μmol/L)	13.3 (4.0 to 127.5)	13.3 (4.0 to 127.5)	13.7 (4.6 to 55.9)	0.614
Albumin (g/L)	42.3 (22.0 to 55.0)	42.5 (26.2 to 55.0)	42.0 (22.0 to 55.0)	0.218
Prothrombin time (s)	11.7 (9.5 to 21.8)	11.7 (9.5 to 21.8)	11.7 (9.9 to 15.8)	0.349
C‐reactive protein (mg/L)	2.78 (0.00 to 269.43)	2.69 (0.00 to 269.43)	3.00 (0.00 to 198.81)	0.404

AFP, alpha‐fetoprotein; ALT, alanine transaminase; AST, aspartate transaminase; MELD, Model for end‐stage liver disease.

### 3.2 Survival data

The last follow‐up date was January 10, 2018. The median follow‐up period was 648 days, ranging from 31 to 1581 days. And the median follow‐up period was 635 days (range, 34‐1581 days) for training group and 666.5 days (range 31‐1454 days) for validation group, respectively. During the follow‐up period, 380 patients were died (38.8%). The 1‐year OS was 75.6% in the primary cohort, 77.0% in training cohort and 72.8% in validation cohort. The 3‐year OS was 53.7%, 54.1% and 54.9%, respectively. There was no significant difference in the OS between training cohort and validation cohort (*P* = 0.780).

### 3.3 Inflammation‐based score system

The following systems were chosen for analyses: platelet‐lymphocyte ratio (PLR), neutrophil‐lymphocyte ratio (NLR), lymphocyte‐monocyte ratio (LMR), CRP ALB ratio (CAR),^9^ the Glasgow Prognostic Score (GPS),^14^ the modified Glasgow Prognostic Score (mGPS)^15^,prognostic nutritional index (PNI)^10^ and Systemic Immune‐Inflammation Index (SII).^16^ The definition and optimal cut‐offs identified by calculating the Youden index was shown in Table [Link], [Link], [Link], [Link]. Most of the patients were of low inflammation score (Table [Link], [Link], [Link], [Link]). The higher inflammation‐based scores were correlated with worse OS for all inflammation‐based scores systems (Table [Link], [Link], [Link], [Link] and Figure [Link], [Link], [Link], [Link]).

For the training cohort, the C‐index and AUC of CAR were, respectively, 0.707 (95% CI, 0.690‐0.725) and 0.728 (95% CI, 0.688‐0.768), which were higher than those of any other score system (Table 2 and Figure [Link], [Link], [Link], [Link]). The CAR was smaller than 0.05 (Score 0) in 291 patients (44.16%), between 0.05 and 0.10 (Score 1) in 110 patients (16.69%) and larger than 0.10 (Score 2) in 258 patients (39.15%), respectively (Table S2). The estimates of OS in 1‐year and 3‐year were worse for the patients with higher CAR scores (Figure S1H, *P < *0.001). For the patients with CAR score 0, 1 and 2, estimated 1‐year OS were 93.7%, 82.3% and 55.5%, and estimated 3‐year survival were 74.3%, 54.6%, and 28.2%, respectively. As described in Methods section, the CAR parameter was selected to build a novel predicting system.

**Table 2:** C‐index and AUC of the Inflammation‐based score systems for training cohort

Inflammation‐based score systems	C‐index	AUC
PLR (0/1)	0.601 (0.586‐0.617)	0.637 (0.592‐0.681)
NLR (0/1)	0.611 (0.595‐0.627)	0.632 (0.587‐0.677)
LMR (0/1)	0.595 (0.579‐0.611)	0.562 (0.517‐0.608)
CAR (0/1/2)	0.707 (0.690‐0.725)	0.728 (0.688‐0.768)
GPS (0/1/2)	0.643 (0.631‐0.656)	0.609 (0.564‐0.653)
mGPS (0/1/2)	0.640 (0.628‐0.653)	0.615 (0.571‐0.659)
PNI (0/1)	0.555 (0.544‐0.565)	0.581 (0.536‐0.626)
SII (0/1)	0.600 (0.583‐0.616)	0.600 (0.556‐0.644)

CAR, C‐reactive protein albumin ratio; GPS, Glasgow Prognostic Score; LMR, lymphocyte‐monocyte ratio; mGPS, the modified Glasgow Prognostic Score; NLR, neutrophil‐lymphocyte ratio; PLR, platelet‐lymphocyte ratio; PNI, prognostic nutritional index; SII, Systemic Immune‐Inflammation Index.

### 3.4 Establishment of the novel system in the primary cohort

Univariate analysis was performed to identify potential correlation between OS and the variables. Ten variables, including BMI, AST, ALT, liver cirrhosis, AFP level, CAR level, tumor size, macrovascular invasion, tumor number, and extra‐hepatic metastases, were associated with OS (Table [Link], [Link], [Link], [Link]). Multivariate analysis identified five variables that were independent risk factors of OS: CAR level, tumor size, macrovascular invasion, tumor number, and extra‐hepatic metastases (Table 3).

**Table 3:** Univariate and multivariate analysis of variables affecting overall survival

Variable	Univariate	Multivariate	
	*P* value	Adjusted HR (95% CI)	*P* value
BMI	0.007		0.529
Extra‐hepatic metastases	<0.001	2.421 (1.746‐3.359)	<0.001
Tumor number	<0.001	1.888 (1.433‐2.487)	<0.001
Tumor size	<0.001	1.781 (1.397‐2.272)	<0.001
Macrovascular invasion	<0.001	1.921 (1.441‐2.561)	<0.001
Liver cirrhosis	<0.001		0.397
AST	<0.001		0.449
ALT	<0.001		0.150
AFP level	<0.001		0.062
CAR level	<0.001	1.640 (1.384‐1.942)	<0.001

AFP, alpha‐fetoprotein; ALT, alanine transaminase; AST, aspartate transaminase; BMI, body mass index; CAR, C‐reactive protein albumin ratio.

**Figure 1:**
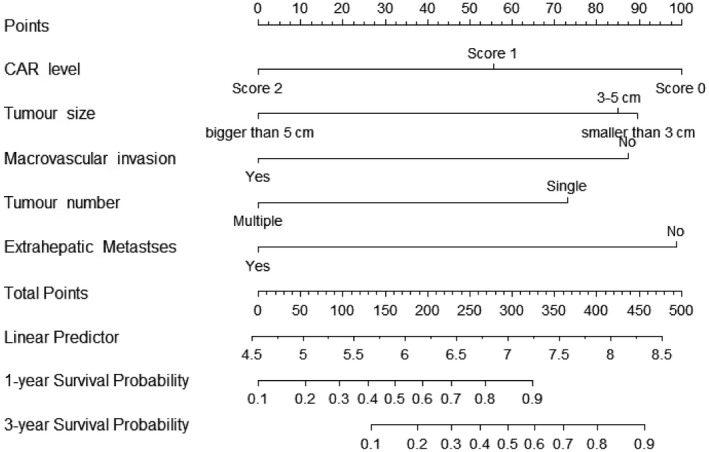
Survival nomogram for hepatocellular carcinoma patients

**Figure 2:**
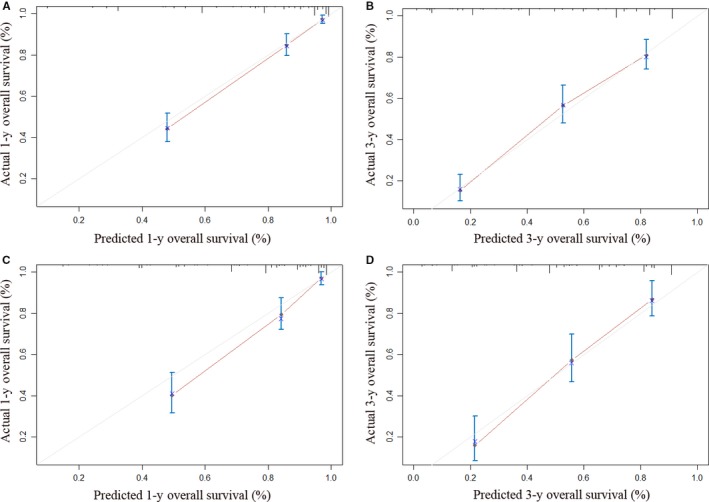
Calibration curve for predicting patient survival. A, At 1 y in the training cohort; B, At 3 y in the training cohort; C, At 1 y in the validation cohort; D, At 3 y in the validation cohort

All significant independent factors identified from multivariate analysis were integrated to build the novel prognostic nomogram (Figure 1). The C‐index for OS prediction was 0.813 (95% CI, 0.789‐0.837). The calibration plot for probability of survival at 1 or 3 years showed a fair agreement between the prediction by nomogram and actual observation (Figure 2A,B).

**Figure 3:**
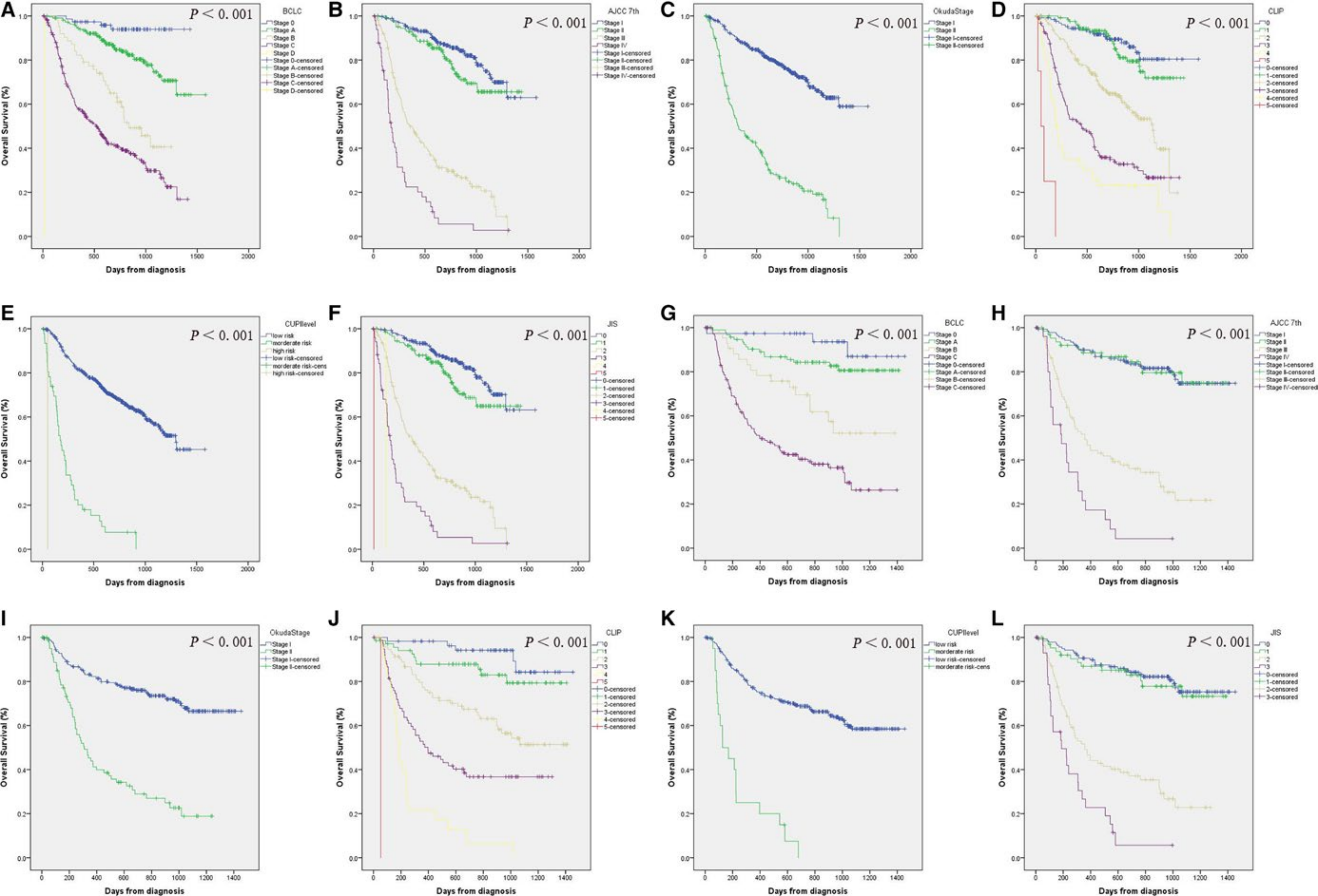
Kaplan‐Meier survival curves of overall survival for different staging systems. A‐F, training cohort; A, Barcelona Clinic Liver Cancer (BCLC); B, American Joint Committee on Cancer (AJCC) 7th edition; C, Okuda stage; D, Cancer of the Liver Italian Program (CLIP); E, Chinese University Prognostic Index (CUPI); F, Japan Integrated Staging Score (JIS); G‐L, validation cohort; G, BCLC; H, AJCC 7th edition; I, Okuda stage; J, CLIP; K, CUPI; L, JIS

Kaplan‐Meier curves were generated for all the conventional staging systems. As shown in Figure 3, nearly all of the curves showed clear different prognostic strata for all the staging system (*P* < 0.001). And the 1‐ and 3‐year OS were also calculated and compared among different strata (Table [Link], [Link], [Link], [Link]). The C‐indexes of the staging system were also calculated. Although most systems showed a C‐index higher than 0.7, the C‐index of CUPI was only 0.585 (95% CI, 0.578‐0.591). Comparing with the conventional staging systems, the nomogram showed a potential high predicting value with a larger C‐index (*P* < 0.001 for all comparison between each conventional staging system and nomogram, Table 4).

**Table 4:** C‐index and 95% CI of the staging systems and nomogram

	Training cohort	Validation cohort	Radical cohort	Palliative cohort
BCLC	0.725 (0.707‐0.743)**	0.715 (0.689‐0.741)**	0.714 (0.688‐0.740)**	0.618 (0.601‐0.635)**
AJCC 7th	0.780 (0.762‐0.797)**	0.744 (0.719‐0.769)**	0.744 (0.719‐0.769)**	0.707 (0.688‐0.726)
Okuda	0.682 (0.669‐0.695)**	0.644 (0.625‐0.663)**	0.606 (0.592‐0.620)**	0.681 (0.663‐0.699)**
CLIP	0.750 (0.732‐0.769)**	0.752 (0.726‐0.779)**	0.721 (0.694‐0.748)**	0.604 (0.588‐0.620)**
CUPI	0.585 (0.578‐0.591)**	0.571 (0.562‐0.580)**	0.522 (0.517‐0.527)**	0.589 (0.579‐0.599)**
JIS	0.776 (0.759‐0.794)**	0.737 (0.712‐0.762)**	0.744 (0.719‐0.769)*	0.689 (0.671‐0.707)**
Nomogram	0.813 (0.789‐0.837)	0.794 (0.756‐0.832)	0.776 (0.729‐0.823)	0.718 (0.688‐0.748)

AJCC 7th, the American Joint Committee on Cancer seventh edition; BCLC, Barcelona Clinic Liver Cancer; CLIP, Cancer of the Liver Italian Program; CUPI, Chinese University Prognostic Index; JIS, Japan Integrated Staging Score.

a *P* < 0.05 when comparing with nomogram.

b *P* < 0.001 when comparing with nomogram

### 3.5 Predicting value in validation cohort

The C‐index of nomogram in the validation cohort was 0.794 (95% CI, 0.756‐0.832), which was higher than any other system (*P* < 0.001 for all comparison between each conventional staging system and nomogram, Table 4). And the calibration plot for probability of survival at 1 or 3 years showed a good correlation between the prediction by nomogram and actual observation (Figure 2C,D). For the patients in the validation cohort, Kaplan‐Meier curves and survival rates were also calculated for all the conventional staging systems. Similar to those in training cohort, different prognostic strata were shown in the curves for all staging systems (Figure 3 and Table [Link], [Link], [Link], [Link]).

### 3.6 Subgroup analysis

Subgroup analysis was performed according to initial treatment. As shown in Table 4, the C‐index of nomogram was 0.776 (95% CI, 0.729‐0.823) in the subgroup of patients received radical treatments. In the subgroup of patients received palliative treatment, the C‐index of nomogram was 0.718 (95% CI, 0.688‐0.748). Comparing with conventional staging systems, the C‐index was significant higher in both groups, except the AJCC 7th system in the palliative group (*P* = 0.364). The calibration plot for probability of survival also showed a good correlation between the prediction by nomogram and actual observation (Figure [Link], [Link], [Link], [Link]).

## 4 DISCUSSION

This study established a novel inflammation‐based predicting system with nomogram based on a prospective cohort of HCC patients. Among a series of inflammation‐based score systems (CAR, PLR, NLR, PMR, GPS, mGPS, PNI, and SII), CAR was selected to construct the novel predicting system because of a higher C‐index and AUC. The other variables, including tumor size, macrovascular invasion, tumor number and extra‐hepatic metastases, were also selected through multivariate analysis. The final nomogram system showed an accurate predicting value. The C‐index of the nomogram was higher (0.813 in the training cohort and 0.794 in the validation cohort), compared with the conventional staging systems. And the calibration curves showed a good correlation between the prediction and actual observation as well.

The inflammation‐based predicting system is of great potential to estimate the prognosis of HCC patients. Previously, several staging systems had been developed for the classification of cancer and selection of treatment options. Although multiple staging systems have been proposed, no consensus has reached on the best system to apply.^17^ In the last few years, investigators have demonstrated that inflammation is a critical accelerator of tumor progression, and the systemic inflammatory response is associated with a poor outcome in patients with malignant tumors, including HCC.^9^, ^11^, ^16^ Even though the mechanism by which systemic inflammation affect the survival was not thoroughly understood, some explanations were proposed. Cancer cells, regarded as exogenous factors, could induce the production of inflammatory cytokines, such as interleukin‐6 (IL‐6), tumor necrosis factor (TNF) and vascular endothelial growth factor (VEGF). The inflammatory cells, and the chemokines, and cytokines that they produce, influence the tumor body, regulate the growth, migration, and differentiation of all cell types in the tumor microenvironment.^18^, ^19^ It could be the theoretical basis of establishing a more effective system predicting the outcome of the HCC patients by developing such system including inflammation‐based factors.

The CAR has the most satisfying prognostic predicting value among systems based on systemic inflammation. Initially, the prognosis value of CAR was found in the outcome of acute exacerbations of chronic disease by Fairclough et al.^20^ And the correlation between CAR and tumor was subsequently evaluated in various studies.^9^, ^21^, ^22^ Kinoshita and his colleagues suggested that CAR may be an independent prognostic marker in patients with HCC, and may have comparable prognostic value with other established inflammation‐based prognostic scores (GPS, mGPS, and NLR).^9^ With a similar result in Kinoshita's study, we proved that the prognostic capacity of CAR was superior to the other inflammation‐based prognostic scores. A few more inflammation‐based prognostic scores were included in our study, the comparison made it clear that CAR might be the optimal choice to establish a predicting system. As emerging studies posted our enhanced concept that inflammation was closely associated with tumor progression, the inclusion of related indicators would improve the rationality of the present staging system. But to date, the inflammation‐based score systems were not widely used in clinical practice independently. Partly because the characteristics relative with tumor burden, such as tumor size, tumor number, macrovascular invasion and extra‐hepatic metastases, were proved to be correlated closely with OS for HCC patients. And the conventional staging systems were largely based on such characteristics. These characteristics were also applied in building nomogram to predicting OS or other outcome for HCC patients in recent years.^23^, ^24^, ^25^ Predicting the OS of HCC patients without considering such characteristics could not be acceptable. Hence, the best policy to improve the existing staging system is to combine the clinical features of the tumor and proper indicators of the systemic inflammation.

In our nomogram, five variants recognized from multivariate analysis were included: tumor size, tumor number, macrovascular invasion, extra‐hepatic metastases and CAR. The variants included were similar with those in AJCC 7th systems. But microvascular invasion was not considered in our study because the assessment of microvascular invasion in patients who did not undertake hepatectomy was unavailable. The AFP level and situation of liver cirrhosis were reported correlated with OS and included in some staging systems.^24^, ^25^ But these two factors were not statistically significant in multivariate analysis.

The system could be applied to optimize clinical practice. Firstly, the CAR level could reflect the systemic inflammation state, which was reported associated with tumor progression. Secondly, for the conventional staging system, the most advanced tumor factor was picked up to predict the OS. For example, a patient with extra‐hepatic metastasis was assigned to the advanced stage, ignoring the liver tumor situation. And the novel system in our study could synthetically calculate all significant characteristics. Thirdly, the nature of nomogram could provide an exact score to predict OS, instead of simple grades, which offer a more powerful and accurate predicting ability. More application of this scoring system was supposed to practice. In clinical trials, this scoring system could be applied to stratify patients into different prognostic level. And the patients suitable for a specific trial could be selected according to the prognostic level. This will make benefit in reduce selection bias. Similarly, this system could potentially select targeted patients for medical therapy and immunotherapy.

Another merit of our system is that it can be applied in patients under different stages. Most of the nomograms reported previously were based on particular patients, such as patients undertaking liver transplantation or hepatectomy.^9^, ^23^, ^26^ No available powerful predicting systems could be commonly applied in all patients receiving different treatments. Similar with the conventional staging systems, the nomogram constructed in our study aimed to universally predict the OS of HCC patients in all stages. The C‐indexes of the nomogram was 0.813 for the training cohort and 0.794 for the validation cohort. In previous studies aimed to develop a nomogram to predict individualized survival risk, Chia‐Yang Hsu reported a nomogram predicting 3‐year OS with a C‐index of 0.71 and Ju‐Hyun Shim reported a nomogram with a C‐index of 0.66.^26^, ^27^ Comparing with the other studies, our nomogram showed a higher C‐index in both the training and validation sets. The possible reason was that our nomogram considered both the systemic inflammation and the tumor situation.

There were some limitations of our studies which should be noticed. Firstly, this nomogram was developed from a single‐center cohort. Even though the data were collected prospectively, multi‐center validation could be more convincing. And further study conducted in western countries is essential to confirm the application worldwide. Secondly, the follow‐up time was not long enough. 5‐year OS was not reached, making the predicting value for the long‐term survival unclear in some extent. Thirdly, the patients included in our study were almost in Child‐Pugh A level, whether this nomogram fit HCC patients with more impaired liver function needs further investigation.

## 5 CONCLUSIONS

The novel inflammation‐based nomogram, developed from prospectively collected data in the present study objectively and accurately predicted the OS of HCC patients. This model provides better prognostic estimates than traditional staging systems.

## CONFLICT OF INTEREST

The authors declare no conflict of interest.
