# Supplementary figures and images for: A novel inflammation‐based nomogram system to predict survival of patients with hepatocellular carcinoma
Source: Cancer Med. 2018 Sep 27;7(10):5027–35. doi: 10.1002/cam4.1787 (PMC6198220; doi:10.1002/cam4.1787)

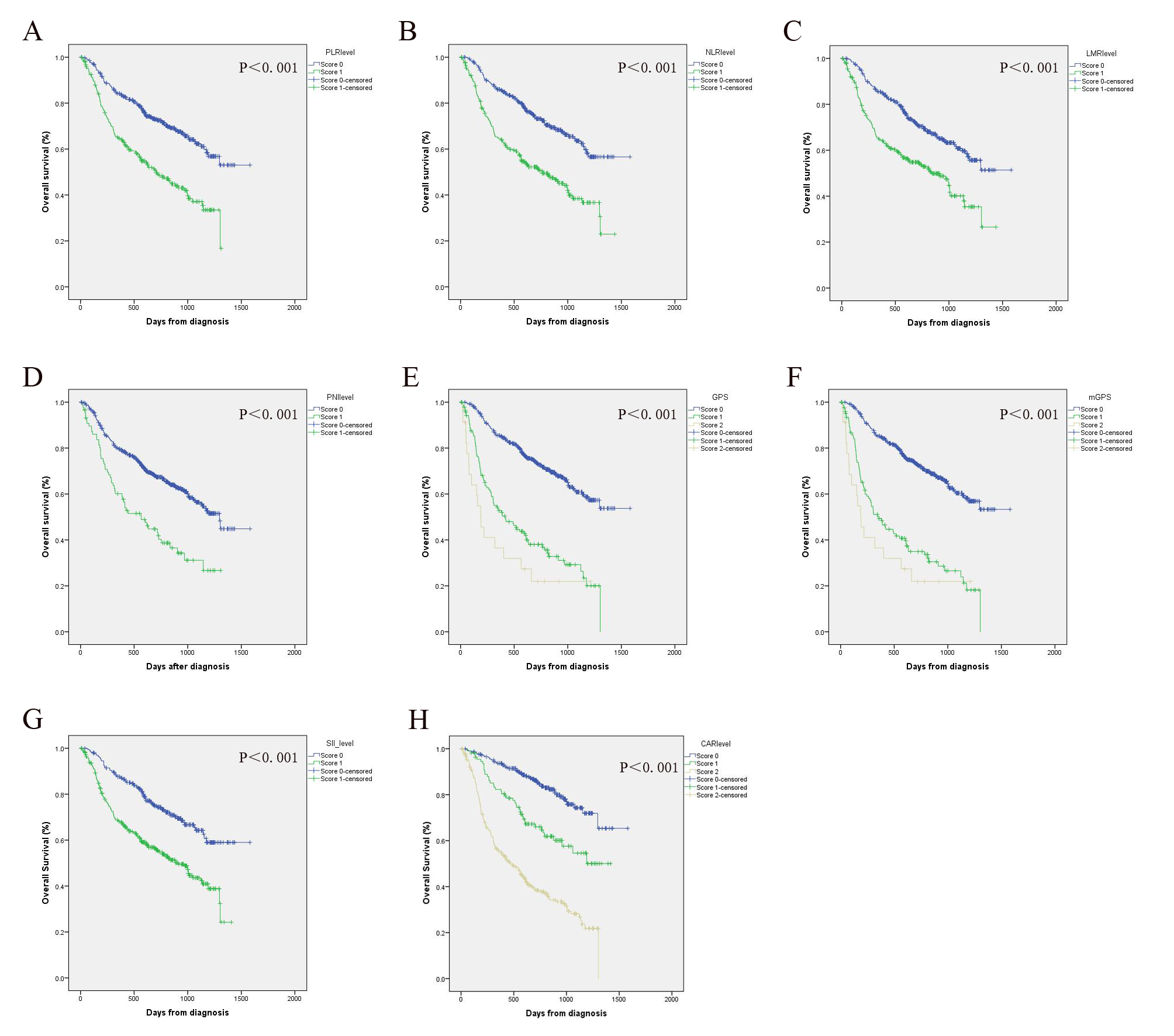

Supplement: Supplementary file 1 [file CAM4-7-5027-s001.tif]

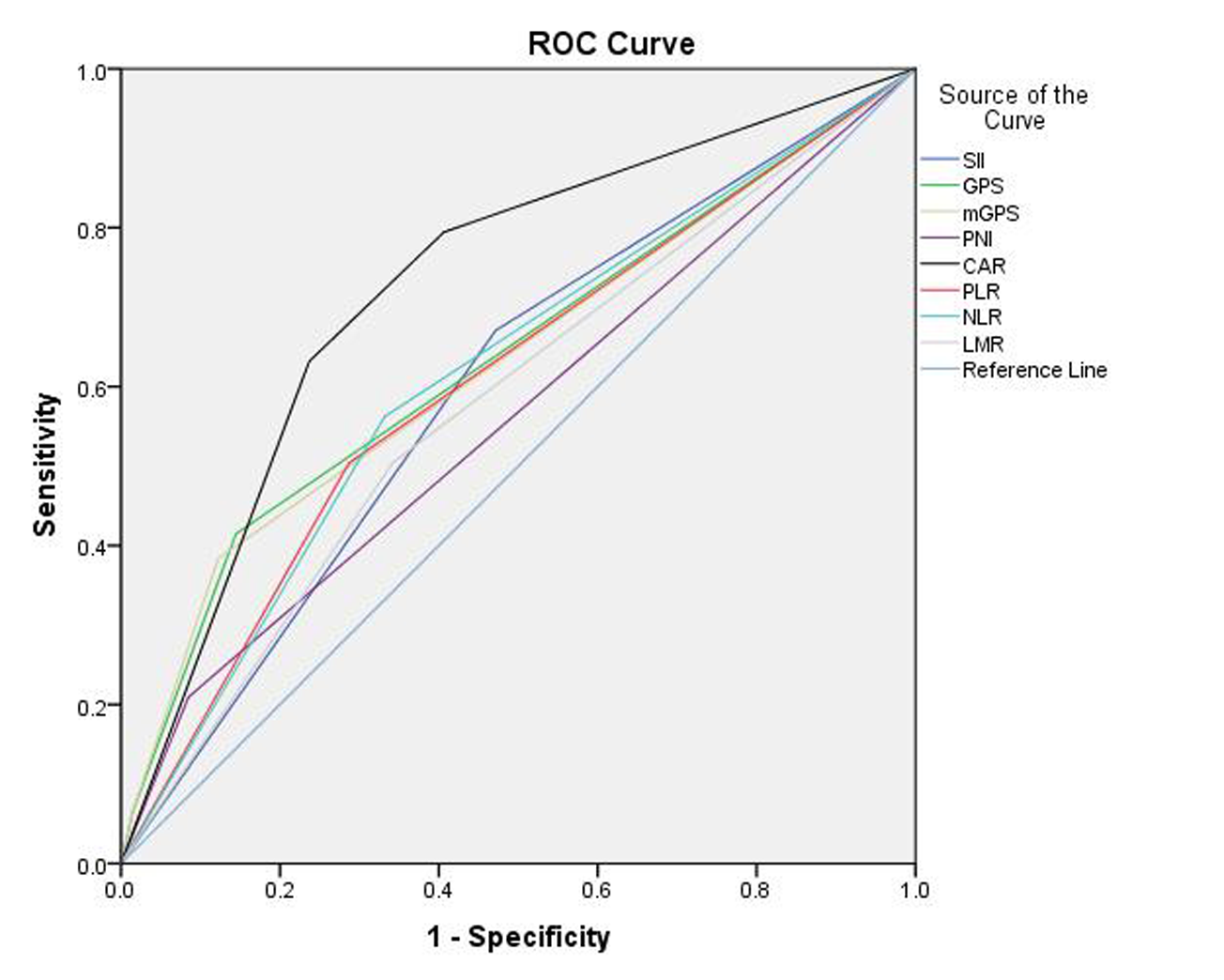

Supplement: Supplementary file 2 [file CAM4-7-5027-s002.tif]

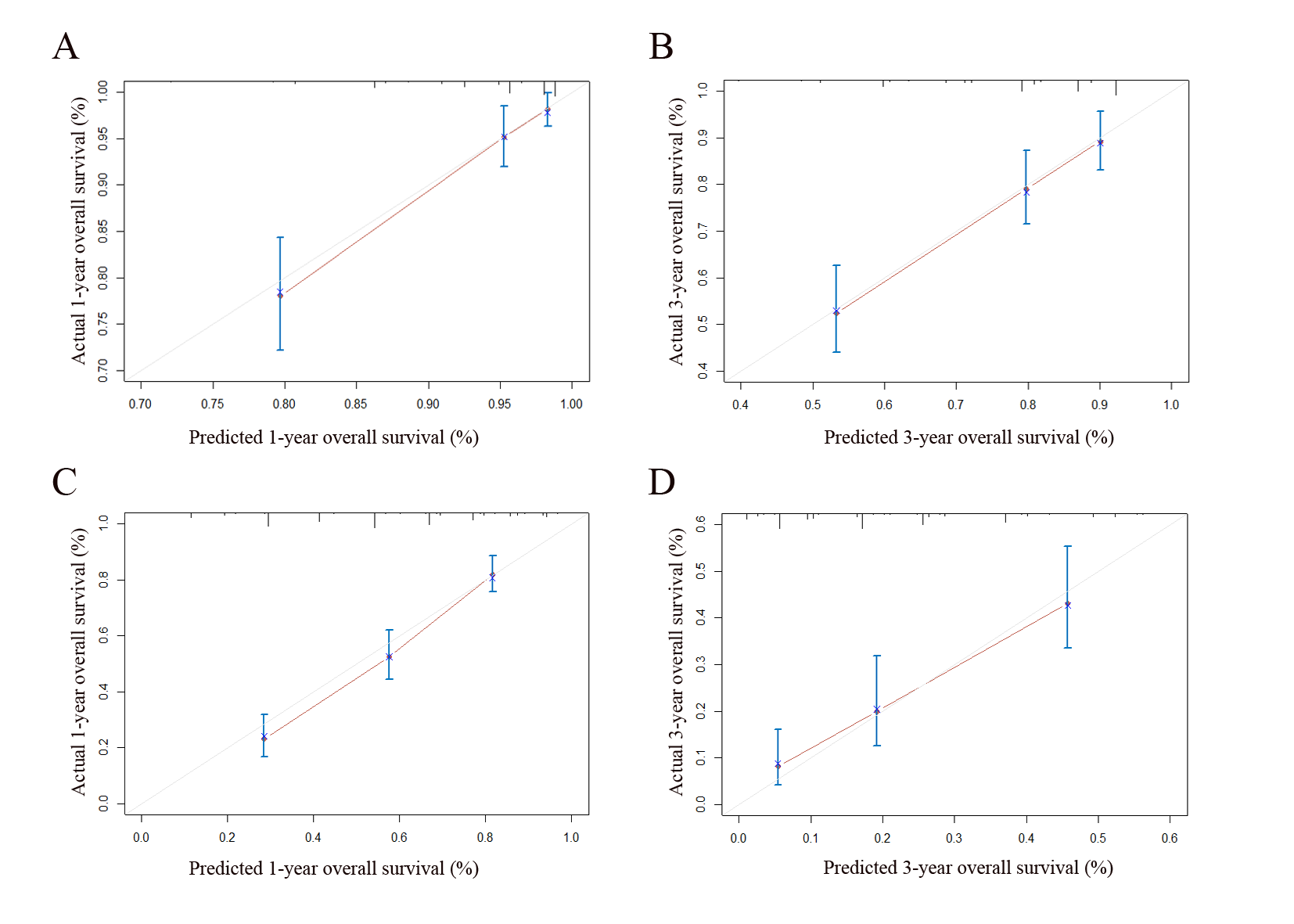

Supplement: Supplementary file 3 [file CAM4-7-5027-s003.tif]
