# Supplementary material for: A novel inflammation‐based nomogram system to predict survival of patients with hepatocellular carcinoma
Source: Cancer Med. 2018 Sep 27;7(10):5027–35. doi: 10.1002/cam4.1787 (PMC6198220; doi:10.1002/cam4.1787)
Supplement: Supplementary file 4 [file CAM4-7-5027-s004.docx]

Supporting materials summary

Supplementary table 1 Definition and cut-offs of the Inflammation-based Score System

Supplementary table 2 Inflammation-based scores and staging systems

Supplementary table 3 The OS in 1-year and 3-year for inflammation-based score systems in training cohort

Supplementary table 4 Univariate analysis of variables affecting OS

Supplementary table 5 The OS in 1-year and 3-year for conventional staging systems

Supplementary figure 1 Kaplan-Meier survival curves of OS for different inflammation-based score systems.

Supplementary figure 2 ROC of different inflammation-based score systems.

Supplementary figure 3 Calibration curve for predicting patient survival. (A) At 1 year in the subgroup of patients with radical treatments; (B) At 3 year in in the subgroup of patients with radical treatments; (C) At 1 year in the subgroup of patients with palliative treatments; (D) At 3 year in the subgroup of patients with palliative treatments;

Supplementary Table 1: Definition and cut-offs of the Inflammation-based Score System

| Inflammaiton-based Score System | Score |
| --- | --- |
| PLR: Platelet Lymphocyte Rratio |  |
| ＜120 | 0 |
| ≥120 | 1 |
| NLR: Neutrophil Lymphocyte Ratio |  |
| ＜2.39 | 0 |
| ≥2.39 | 1 |
| LMR: Lymphocyte Monocyte Ratio |  |
| ≥3.27 | 0 |
| ＜3.27 | 1 |
| CAR: CRP/ALB |  |
| ＜0.05 | 0 |
| 0.05-0.10 | 1 |
| ≥0.10 | 2 |
| GPS： The Glasgow Prognostic Score |  |
| CRP≤10mg/L and ALB≥35g/L | 0 |
| CRP≤10mg/L and ALB＜35g/L | 1 |
| CRP＞10mg/L and ALB≥35g/L | 1 |
| CRP＞10mg/L and ALB＜35g/L | 2 |
| mGPS： The Modified Glasgow Prognostic Score |  |
| CRP≤10mg/L and ALB≥35g/L | 0 |
| CRP≤10mg/L and ALB＜35g/L | 0 |
| CRP＞10mg/L and ALB≥35g/L | 1 |
| CRP＞10mg/L and ALB＜35g/L | 2 |
| PNI: Prognostic Nutritional Index |  |
| ALB (g/L) * lymphocyte count * 10^9^/L ≥45 | 0 |
| ALB (g/L) * lymphocyte count * 10^9^/L＜45 | 1 |
| SII：Systemic Immune-Inflammation Index |  |
| Platelet*Neutrophil/Lymphocyte＜330 | 0 |
| Platelet*Neutrophil/Lymphocyte≥330 | 1 |

Supplementary table 2 Inflammation-based scores and staging systems

| Characteristic | Primary cohort | Training group | Validation group | *P* Value |
| --- | --- | --- | --- | --- |
| PLR (0/1) | 621/358 | 414/245 | 207/113 | 0.570 |
| NLR (0/1) | 570/409 | 381/278 | 189/131 | 0.710 |
| LMR (0/1) | 583/396 | 392/267 | 191/129 | 0.951 |
| CAR (0/1/2) | 423/166/390 | 291/110/258 | 132/56/132 | 0.690 |
| GPS (0/1/2) | 730/212/37 | 494/142/23 | 236/70/14 | 0.779 |
| mGPS (0/1/2) | 755/187/37 | 511/125/23 | 244/62/14 | 0.774 |
| PNI (0/1) | 847/132 | 571/88 | 276/44 | 0.865 |
| SII(0/1) | 441/538 | 297/362 | 144/176 | 0.984 |
| AJCC 7th (Ⅰ/Ⅱ/Ⅲ/Ⅳ) | 426/210/265/78 | 286/145/177/51 | 140/65/88/27 | 0.928 |
| BCLC (0/A/B/C/D) | 113/316/105/444/1 | 75/221/62/300/1 | 38/95/43/144/0 | 0.309 |
| Okuda (Ⅰ/Ⅱ) | 714/265 | 477/182 | 237/83 | 0.579 |
| CLIP(0/1/2/3/4/5) | 184/196/289/22/73/5 | 127/127/195/156/50/4 | 57/69/94/76/23/1 | 0.943 |
| CUPI (Low/Moderate/High risk) | 911/67/1 | 611/47/1 | 300/20/0 | 0.686 |
| JIS(0/1/2/3/4/5) | 423/211/260/83/1/1 | 284/146/174/53/1/1 | 139/65/86/30/0/0 | 0.878 |

Supplementary table 3 The OS in 1-year and 3-year for inflammation-based score systems in training cohort

|  | 1-year OS | 3-year OS | P value |
| --- | --- | --- | --- |
| PLR |  |  | ＜0.001 |
| Score 0 | 84.2% | 62.3% |  |
| Score 1 | 64.6% | 33.5% |  |
| NLR |  |  | ＜0.001 |
| Score 0 | 86.0% | 63.6% |  |
| Score 1 | 64.5% | 38.4% |  |
| LMR |  |  | ＜0.001 |
| Score 0 | 85.4% | 60.8% |  |
| Score 1 | 64.4% | 40.1% |  |
| GPS |  |  | ＜0.001 |
| Score 0 | 85.5% | 60.9% |  |
| Score 1 | 52.8% | 29.3% |  |
| Score 2 | 36.5% | 21.9% |  |
| mGPS |  |  | ＜0.001 |
| Score 0 | 85.2% | 60.4% |  |
| Score 1 | 49.3% | 26.5% |  |
| Score 2 | 36.5% | 21.9% |  |
| PNI |  |  | ＜0.001 |
| Score 0 | 79.6% | 56.4% |  |
| Score 1 | 60.1% | 31.1% |  |
| SII |  |  | ＜0.001 |
| Score 0 | 87.6% | 64.2% |  |
| Score 1 | 68.2% | 43.6% |  |
| CAR |  |  | ＜0.001 |
| Score 0 | 93.7% | 74.3% |  |
| Score 1 | 82.3% | 54.6% |  |
| Score 2 | 55.5% | 28.2% |  |

Supplementary table 4 Univariate analysis of variables affecting OS

| Variable | *P* value | Adjusted HR (95% CI) |
| --- | --- | --- |
| Age (≤65 vs. ＞65 years) | 0.073 | 0.712 (0.491-1.031) |
| Gender (Male vs. female) | 0.113 | 1.395 (0.923-2.108) |
| BMI | 0.007* | 0.945 (0.908-0.984) |
| ALT (≤40 vs. ＞40U/L) | <0.001* | 1.662 (1.298-2.129) |
| AST (≤35 vs. ＞35U/L) | <0.001* | 2.738 (2.057-3.644) |
| TBIL (≤20 vs. ＞20umol/L) | 0.095 | 1.303 (0.954-1.778) |
| PT (≤13.5 vs. ＞13.5s) | 0.110 | 1.400 (0.926-2.116) |
| AFP (≤400 vs. ＞400ng/ml) | <0.001* | 1.940 (1.517-2.481) |
| HBV (No vs. Yes) | 0.896 | 1.027 (0.680-1.553) |
| HCV (No vs. Yes) | 0.252 | 0.443 (0.110-1.783) |
| MELD | 0.282 | 1.022 (0.981-1.065) |
| Extra-hepatic metastases (No vs. Yes) | <0.001* | 5.398 (3.962-7.354) |
| Tumour Number (Single vs. multipul) | <0.001* | 3.690 (2.867-4.748) |
| Macrovascular Invasion (No vs. Yes) | <0.001* | 4.454 (3.413-5.812) |
| Tumour Size level | <0.001* | 3.035 (2.430-3.791) |
| Liver Cirrhosis(No vs. Yes) | <0.001* | 0.564 (0.442-0.721) |
| CAR level | <0.001* | 2.367 (2.032-2.756) |

*P＜0.05

Supplementary table 5 The OS in 1-year and 3-year for conventional staging systems

|  | Training | | | Validation | | |
| --- | --- | --- | --- | --- | --- | --- |
|  | 1-year OS | 3-year OS | P value | 1-year OS | 3-year OS | P value |
| BCLC |  |  | ＜0.001 |  |  | ＜0.001 |
| Stage 0 | 97.3% | 94.0% |  | 97.4% | 86.9% |  |
| Stage A | 94.0% | 74.6% |  | 90.2% | 80.6% |  |
| Stage B | 82.3% | 40.6% |  | 80.7% | 52.2% |  |
| Stage C | 57.7% | - |  | 51.9% | 26.3% |  |
| Stage D | - | - |  | - | - |  |
| AJCC 7^th^ |  |  | ＜0.001 |  |  | ＜0.001 |
| Stage I | 94.3% | 75.0% |  | 89.9% | 74.7% |  |
| Stage II | 93.0% | 65.6% |  | 88.6% | 74.9% |  |
| Stage III | 49.9% | 20.7% |  | 49.2% | 21.7% |  |
| Stage IV | 22.4% | 2.8% |  | 17.2% | - |  |
| Okuda |  |  | ＜0.001 |  |  | ＜0.001 |
| Stage I | 87.9% | 66.1% |  | 82.3% | 66.5% |  |
| Stage II | 47.9% | 19.2% |  | 43.9% | 18.9% |  |
| CLIP |  |  | ＜0.001 |  |  | ＜0.001 |
| Stage 0 | 94.4% | 80.4% |  | 98.3% | 84.2% |  |
| Stage 1 | 97.6% | 71.9% |  | 87.9% | 79.4% |  |
| Stage 2 | 82.7% | 53.3% |  | 77.3% | 51.4% |  |
| Stage 3 | 53.1% | 26.7% |  | 51.5% | 36.7% |  |
| Stage 4 | 34.8% | 23.0% |  | 21.7% | #N/A |  |
| Stage 5 | - | - |  | - | - |  |
| CUPI |  |  | ＜0.001 |  |  | ＜0.001 |
| low risk | 81.4% | 56.8% |  | 76.1% | 58.5% |  |
| morderate risk | 20.2% | - |  | 25.0% | - |  |
| high risk | - | - |  | - | - |  |
| JIS |  |  | ＜0.001 |  |  | ＜0.001 |
| Stage 0 | 94.6% | 75.2% |  | 90.6% | 75.3% |  |
| Stage 1 | 92.4% | 65.0% |  | 86.9% | 73.2% |  |
| Stage 2 | 51.3% | 21.6% |  | 49.2% | 22.9% |  |
| Stage 3 | 21.5% | 2.7% |  | 22.9% | - |  |
| Stage 4 | - | - |  | - | - |  |
| Stage 5 | - | - |  | - | - |  |

Supplementary Figure 1 Kaplan-Meier survival curves of OS for different inflammation-based score systems: A, PLR; B, NLR; C, LMR; D, PNI; E, GPS; F, mGPS; G, SII; H, CAR.

Supplementary figure 2 ROC of different inflammation-based score systems.

Supplementary figure 3 Calibration curve for predicting patient survival. (A) At 1 year in the subgroup of patients with radical treatments; (B) At 3 year in in the subgroup of patients with radical treatments; (C) At 1 year in the subgroup of patients with palliative treatments; (D) At 3 year in the subgroup of patients with palliative treatments;
